# Itch Relief by Mirror Scratching. A Psychophysical Study

**DOI:** 10.1371/journal.pone.0082756

**Published:** 2013-12-26

**Authors:** Christoph Helmchen, Carina Palzer, Thomas F. Münte, Silke Anders, Andreas Sprenger

**Affiliations:** Department of Neurology, University of Luebeck, Luebeck, Germany; Barrow Neurological Institute, United States of America

## Abstract

**Objective:**

The goal of this study was to test whether central mechanisms of scratching-induced itch attenuation can be activated by scratching the limb contralateral to the itching limb when the participant is made to visually perceive the non-itching limb as the itching limb by means of mirror images.

**Methods:**

Healthy participants were asked to assess the intensity of an experimentally induced itch at their right forearm while they observed externally guided scratch movements either at their right (itching) or left (non-itching) forearm which were either mirrored or not mirrored. In the first experiment, a mirror placed between the participant’s forearms was used to create the visual illusion that the participant’s itching (right) forearm was being scratched while in fact the non-itching (left) forearm was scratched. To control visibility of the left (non-mirrored) forearm, a second experiment was performed in which unflipped and flipped real-time video displays of the participant’s forearms were used to create experimental conditions in which the participant visually perceived scratching either on one forearm only, on both forearms, or no scratching at all.

**Results:**

In both experiments, scratching the non-itching limb attenuated perceived itch intensity significantly and selectively in the mirror condition, i.e., when the non-itching forearm was visually perceived as the itching limb.

**Discussion:**

These data provide evidence that the visual illusion that an itching limb is being scratched while in fact the non-itching limb contralateral to the itching limb is scratched, can lead to significant itch relief. This effect might be due to a transient illusionary intersensory perceptual congruency of visual, tactile and pruriceptive signals. “Mirror scratching” might provide an alternative treatment to reduce itch perception in focal skin diseases with persistent pruritus without causing additional harm to the affected skin and might therefore have significant clinical impact.

## Introduction

Itch can be defined as an unpleasant sensation that provokes the desire to scratch the itching site. Itch is attenuated by scratching. Many inflammatory skin diseases, e.g. atopic eczema, elicit an itch sensation [1] but patients must not scratch the itching skin rashes as skin inflammation might deteriorate. Unfortunately, sustained itch relief is not always achieved by standard drug treatment. Thus, there is a strong need for additional interventions in persistent pruritus. 

Histamine reliably elicits itch and a flare by axon reflexes and is therefore used in many experimental human models of itch. In inflammatory skin lesions, histamine is physiologically released by mast cells and activates unmyelinated peripheral C-fibers and spinothalamic lamina I neurons [2-4]. Via spinothalamic afferents these signals are transmitted to brain regions that encode location and intensity of somatosensory sensations , i.e., the primary and secondary somatosensory cortex [5], and valence, i.e., insula and anterior cingulate cortex (ACC) [6].

Under normal conditions, scratching immediately attenuates itch. It has been proposed that scratching-related itch relief is best explained by spinal and supraspinal interactions rather than peripheral receptor-mediated mechanisms [7,8]. For example, excitation of spinothalamic tract neurons by stimulation of the primary afferents by histamine is attenuated by scratching [9]. Itch relief can also be obtained by scratching sites remote from the itching site [10,11] suggesting that central mechanisms may be involved in the control of itch. Scratching does not need to be conducted by oneself but can also alleviate itch when performed by somebody else at the itching or a remote skin area [7]. 

Driven by clinical demands we sought to establish an experimental condition in which the participant perceives a visible tactile manipulation (scratching) of the non-itching limb to occur on the affected limb. This idea has also recently been proposed in an abstract on phantom itch patients [12]. Altschuler and Scott observed that some patients with itch in a phantom limb noticed phantom itch relief by watching the reflection of scratching on the corresponding intact limb in a mirror [13]. Research in recent years has indicated that multisensory integration can lead to illusionary perceptions in situations that do not normally occur in real-life [14]. For example, observing a mirror image of one’s own limb can lead to the illusionary perception that the mirrored limb is one’s own contralateral limb. A mirror box, placed vertically on the table in front of a subject’s hand, has been used to elicit synaesthesia [15]. When amputees [16] or stroke patients [17] observe their intact limb in a mirror box that is carefully placed parallel to their phantom or paretic forearm this can lead to the illusionary perception that their phantom hand has been resurrected or that their paretic limb is moving [18]. Mirror visual feedback had also been applied to relieve pain in complex regional pain syndrome (CRPS) type 1 [19]. Vision and touch may interact in a way that objects viewed in a mirror are recoded as originating from a location within peripersonal space [20]. Perception in such visuo-tactile conflicts seems to be dominated by visual cues [14]. These examples show that under some conditions, the brain can be “fooled” by multisensory stimulation in a way that stimulations are perceived that do not actually exist. This may elicit visuo- tactile illusions with regard to a person’s self-body schema [14,18,21]. 

In the current study we sought to extend these findings to the perception of itch attenuation. Unlike mirror visual feedback therapy in motor recovery (e.g. stroke patients) we did not try to elicit the visual impression of bimanual movements but referred sensations in the mirrored forearm. Mirror elicited sensations felt on skin sites which are not physically stimulated, i.e., referred sensations, have been shown in several patient groups, e.g. with stroke [22], CRPS [23], and patients with anesthetic limbs [24]. If CRPS type 1 patients observed tactile stimulations of the mirrored image of their unaffected hand in the mirror they felt allodynia on their affected hand [23]. Stimulation of the unaffected limb elicited referred sensation in the affected limb. In contrast to this aversive allodynia we were looking for an itch-attenuating referred sensation. 

We hypothesized that itch relief can be obtained by scratching the limb contralateral to the itching limb if the subject is made to visually perceive the non-itching limb as the itching limb by means of a mirror image. To test this hypothesis, we asked healthy participants to rate the perceived intensity of an experimentally induced, histamine-associated itch before and after they observed externally guided scratch movements either at their itching or their non-itching forearm. Two different experimental approaches were used. 

In the first (mirror) experiment, the visual illusion that the participant’s itching (right) forearm was being scratched [while in fact the participant’s non-itching (left) forearm was being scratched] was elicited by means of a mirror, placed in between the participant’s left and right forearm. In the mirror experiment, the participant was instructed to look into the mirror. While this design has a simple and easy-to-implement experimental set-up that makes it suitable for clinical applications, visibility of the non-itching (left) forearm in the mirror condition is not completely controlled. Thus, in order to rule out that any itch attenuation observed in the mirror condition was induced by visual perception of scratch movements on the mirrored and the real left forearm, we run a second (video) experiment. In the latter, unflipped and flipped real-time video displays of the participant’s forearms were used to create experimental conditions in which the participant visually perceived scratching either on one forearm only, both forearms, or no scratching at all. Very recently, video-mediated mirroring of hands had been shown to induce referred sensations equally powerful compared with mirror reflections [25]. We will show that “mirror scratching”, i.e., the visual illusion that an itching limb is being scratched while in fact the non-itching limb contralateral to the itching limb is scratched, can - at least partially - attenuate itch.

## Methods

### Participants

**Figure 1:**
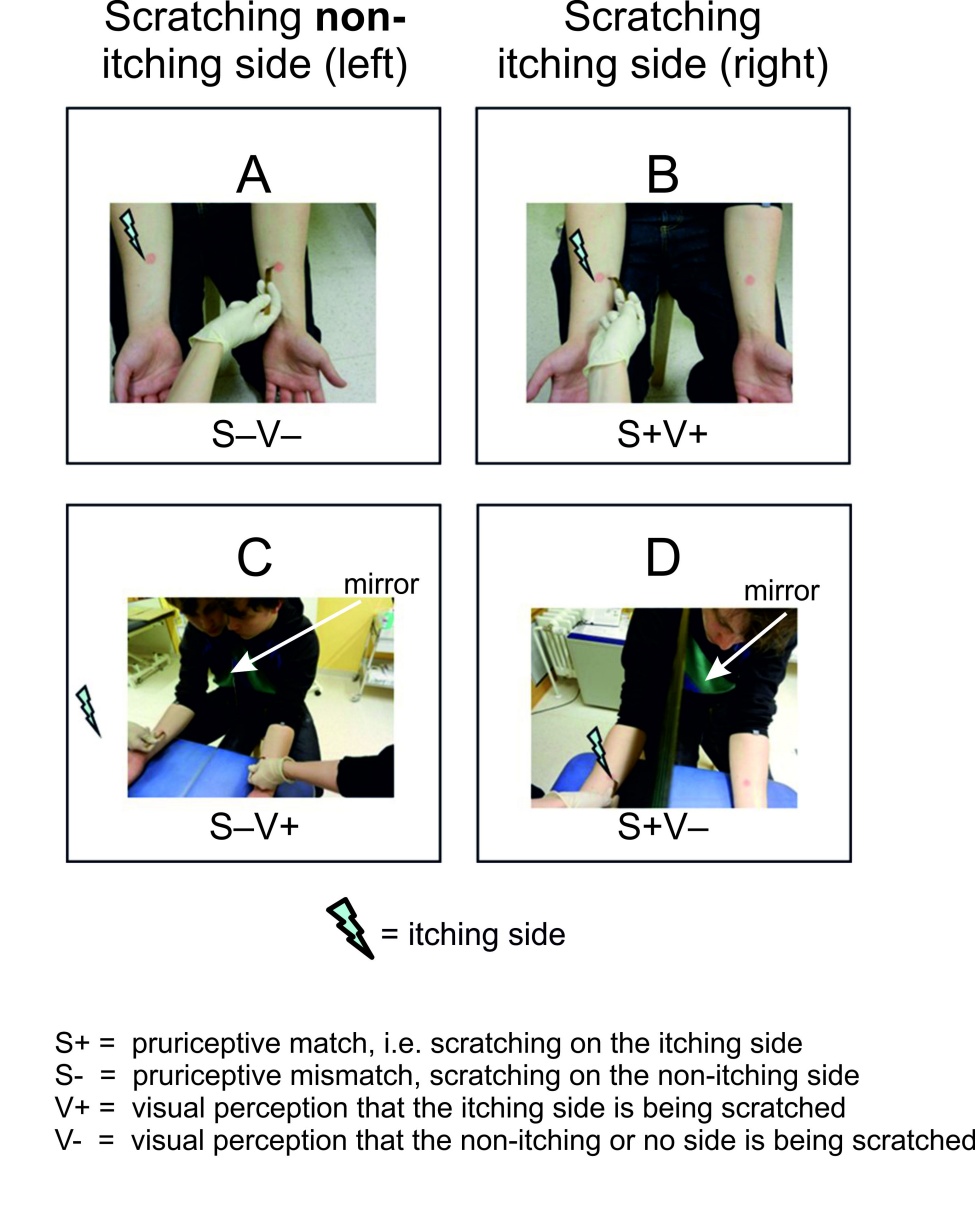
Design of the mirror experiment. The photos show the four conditions of the 2 x 2 factorial design. The two conditions (A,B) with direct view on both forearms are shown in the top row. The two mirror conditions (bottom row) in which the participant visually perceives the non-itching (left) forearm in place of the itching (right) forearm (C) and in which the subject’s view on the scratching of the itching (right) forearm is prevented by the mirror (D). The injection site is masked by red color patches at homologue skin sites on both forearms to prevent visual identification of the itching forearm. The lightning symbol indicates the itching (right) forearm. S-: scratching at the non-itching (left) forearm; S+: scratching at the itching (right) forearm; V-: visual percept that the non-itching (left) forearm is being scratched; V+: visual percept that the itching (right) forearm is being scratched.

Twenty-six male right-handed healthy volunteers [age: 26.5 ±4.5 (SD), range: 19-38 years] participated in the study. One participant was excluded from the analysis, because of large differences in itch intensity ratings between trials (> 3 standard deviations). Five additional participants were excluded because baseline itch ratings did not exceed 15 percent of maximal conceivable, unbearable itch intensity (measured by numeric rating scale, NRS, or visual analogue scale, VAS). All remaining participants participated in both experiments. None of them had a history of spontaneous itching, allergy or inflammatory skin disease including atopic eczema, or symptoms and signs of peripheral or central neurological conditions. The study was approved by the Ethics Committee of the University of Lübeck and was conducted in accordance with the declaration of Helsinki. All participants gave written informed consent before participation. The participant shown in Figure 1 gave written informed consent for publication of the photograph as outlined in the PLOS One consent form.

### Itch induction

Histamine-dihydrochloride (0.03 ml, 1%) was injected (BD Microlance 3 skin needles) into the dermal-epidermal junction of the volar aspect of the right forearm where itch-sensitive C-fibers terminate [26]. This elicited a red spot (diameter: 1.5 cm) around the injection side. To prevent visual identification of the itching side, both forearms were labeled with red color making them visually indistinguishable. After an average latency of 25 sec, an itch sensation without pain sensation developed, gradually increased to reach a maximum at approximately 60 sec after the injection and remained stable for at least 5 minutes before it started to decrease. No participant reported itch more than 12 minutes after the injection. In the first experiment, histamine injection and scratching procedures were performed twice in two identical trials for each experimental condition, separated by a 20 min interval during which itch perception ceased. The second injection was given 2 cm distal to the first one. Data were pooled across the two trials of each condition unless they differed significantly from each other. 

### Scratch procedure

Scratching was applied on the participant’s right or left forearm by the investigator by using an L-shaped bendable copper sheet (thickness: 1mm; width: 10mm; length: 130mm) [7]. Scratching was performed with the buckled smaller end with rounded edges using a force that slightly bended the copper sheet. This exerted a force of 100 ±5 g to the skin. The experimenter took care to conduct 6 even strokes of 6 cm length (each lasting 2.5s) immediately adjacent to the injected site (or the corresponding area on the contralateral forearm). Scratching was trained prior to the experiment and controlled by a digital balance scale. Scratching was not applied directly to the site of histamine injection in order to prevent peripheral interactions at the terminals of itch fibers, instead scratching movements were delivered to a site approximately 2 cm lateral to the histamine injection site or the homologous site on the contralateral, non-itching forearm. 

### Assessment of perceived itch intensity before and after scratching

Itch intensity was rated by each participant before (T0, 60 seconds after injection) and immediately after scratching (T1, within 5 seconds after scratching) using both a numeric (NRS) and visual analogue scale (VAS) [27]. The scales ranged from 0 (no itch) to 100 (maximal conceivable, unbearable itch intensity with an excessive urge to stop the experiment). Participants were told that 10/100 represented a just noticeable itch, 30/100 an annoying itch with the beginning urge to scratch, 70/100 an imperative, still tolerable urge to scratch, and 100/100 required immediate test stop unless scratching is provided. As itch intensity before scratching (T0) was rated differently between participants we used the *change* in itch intensity from T0 to T1 (rating difference, RD), i.e., differences between ratings (T0-T1) rather than relative difference values (in %) entered pre-post data in the result section. The next scratching procedure according to the experimental design (see conditions A-D in experiment 1 and a-h in experiment 2) was performed after 30 sec, which was the interval after which itch intensity reached its maximum again. 

### Study Design

#### Experiment 1: Mirror Experiment

In the first experiment, we used a 2 x 2 design with factors actual side of scratching [S+ = scratching of the itching (right) forearm, S- =scratching of the non-itching (left) side] and visually perceived side of scratching [V+] = visual perception that the itching side is being scratched; V- = visual perception that the non-itching left forearm or no side is being scratched). The two mirror conditions [S+V-] and [S-V+] were created by using a large mirror which was placed vertically between the forearms of the participant. He was instructed to look into the mirror which allowed him to see the mirrored left forearm in the place of the right forearm without seeing the left forearm directly. Sight of the right (itching) forearm was completely prevented by the mirror (Figure 1C,D).The condition [S-V+] Condition (C) was critical in this experiment as it was intended to elicit itch relief by making the participant visually perceive the scratched non-itching (left) forearm as his itching (right), forearm. The three other conditions (A, B, D) served as control conditions. They controlled the perceived effect of itch attenuation by scratching the itching right forearm (B) or the non-itching left forearm (A). Condition (D) controlled for itch attenuation by scratching the itching but not visible forearm. This elicited a visuo-tactile mismatch as the perceived right forearm (mirrored left forearm) was scratched without seeing it. Visual cues of the histamine-induced inflammatory reaction should have been prevented by masking the inflammation by red colored areas looking alike on both forearms.

Please note that the sequence of conditions could not be fully randomized because the mirror needed to be adjusted for the conditions [S+V-] and [S-V+]. Thus, only the order of the non-mirrored conditions [S+V+] and [S-V-] and the order of the mirror conditions [S+V-] and [S-V+] were randomized. 

#### Experiment 2: Video experiment

**Figure 2:**
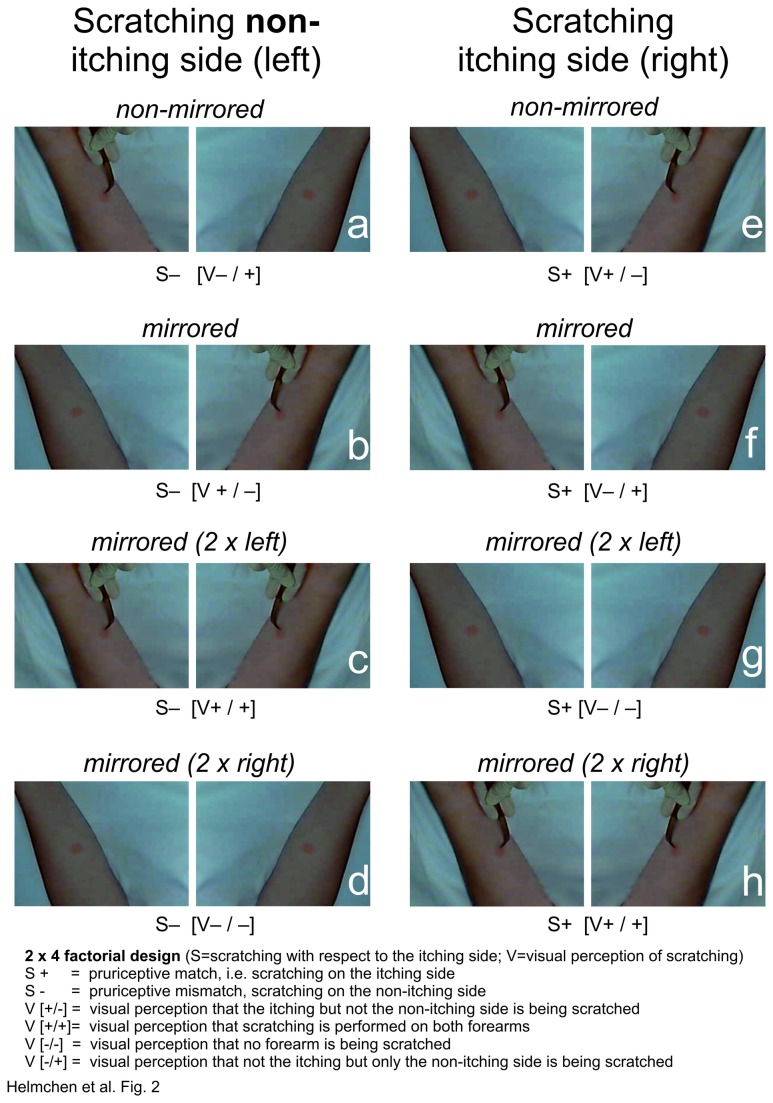
Design of the video experiment. The subject’s view on the real-time video displays in the eight different experimental conditions is shown. As in the mirror experiment, the injection site is masked by red color patches at homologue skin sides on both forearms to prevent visual identification of the itching forearm. Scratching side is indicated by [S-] at the non-itching (left) forearm and by [S+], at the itching (right) forearm. The visual percept that scratching is applied to the non-itching (left) forearm only is indicated [V-/+]; to the itching (right) forearm only [V+/-], to both forearms [V+/+], or visual percept that no scratching is applied [V-/-] (see also Table 1). In the case of two visible forearms, one forearm is flipped (mirrored), the other is visible at it is normally seen.

The second experiment was run to control the participants’ visual perception more closely than it was possible in the first experiment. Note that although participants in the mirror condition of the first experiment were instructed to look into the mirror while being scratched, they might have seen their left forearm both in the mirror and on the table. This might have led to the visual perception of being scratched at the mirrored and the real forearm. In order to control the visual perception of the non-itching (left) forearm in mirror conditions, we performed a second experiment in which we showed the participant flipped and unflipped real-time video displays of either forearm. This created a total of eight experimental conditions (2 x 2 x 2), four in which the itching forearm was being scratched (S+) and four in which the non-itching forearm was being scratched (S-). In two of these four conditions, the *itching* (right) forearm was visually perceived by the participant as being scratched (V+), in the other two the itching forearm was visually perceived as not being scratched (V-). Finally, the *non-itching* (left) forearm was also indicated as being scratched (/+) or being not scratched (/-). The eight experimental conditions (S+ [V+/-]; S+ [V-/+]; S+ [V+/+]; S+ [V-/-]; S- [V+/-]; S- [V-/+]; S- [V+/+]; S- [V-/-]) are shown in Figure 2. Table 1 illustrates each experimental condition (a-h) in Figure 2 as both are presented in the same order to make comparisons easier. According to out hypothesis, the critical condition in this experiment was the condition [S-] [V+/-], which was intended to create the visual illusion that the participant’s itching (right) forearm was being scratched while in fact the non-itching (left) forearm was scratched (similar to condition S- [V+] in the first experiment). Notably, this condition [S-] [V+/-] had the additional constraint that scratching of the non-itching forearm was not seen by the participant. The order of conditions was pseudorandomized, with 4 repetitions of each experimental condition. The experiment was run in four blocks, each consisting of 8 trials. 

**Table 1:** Experimental conditions in the video experiment showing mirrored and non-mirrored forearms while scratching of the non-itching (left, S-) or the itching (right, S+) side.

Scratching **non-itching** (left) side (S-)	Scratching **itching** (right) side (S+)
a)Both arms are not mirrored	e) Both arms are not mirrored
→both arms are shown (unflipped)	→ both arms are shown (unflipped)
→scratching on the left arm visible [V-/+]	→ scratching on right arm visible [V+/-]
b)Both arms are mirrored	f) Both arms are mirrored
→both arms are shown (flipped)	→ both arms are shown (flipped)
→scratching visible on right arm [V+/-]	→ scratching on left arm visible [V-/+]
c) Only the left arm is mirrored	g) Only the left arm is mirrored
→ left arm is shown twice (flipped and non-flipped)	→ left arm is shown twice (flipped and non-flipped)
→ scratching of both arms visible [V+/+]	→ no scratching visible [V-/-]
d) Only the right arm is mirrored	h) Only the right arm is mirrored
→ right arm is shown twice (flipped and non-flipped)	→ right arm is shown twice (flipped and non-flipped)
→ no scratching visible [V-/-]	→ scratching of both arms visible [V+/+]

During the experiment, participants lay supine on a comfortable bed and observed their forearms online on a monitor which was fixed 1 m in front of the participant’s head. Scratching by the investigator was grabbed by a camera (Logitech^®^ C310) which presented real-time displays (latency < 50ms) on the monitor screen by a custom program written in Matlab® (R2012a, The Mathworks, Natick/MA). Due to the very slow scratch movements there was no visible delay between actual movement and the visual display on the monitor. The participant’s field of view was restrained to the monitor screen by a surrounding black curtain (black chamber) preventing penetration of light and other visual cues. Note that the participant’s field of view covered only the lower forearms (without hands, see Figure 2) making it virtually impossible to distinguish them.

### Statistical analysis

Statistical analyses were performed with SPSS 20.0 (IBM Inc. NY/USA). Rating differences (RD) on VAS and NRS were computed within subjects. RD data were regarded as ordinal data. For comparison of two conditions, Wilcoxon-Test was used; in one-factorial analyses Friedman-Test was performed. For multifactorial comparisons RD data were transformed into ranks and analyses of variance (ANOVAs) were performed on rank-transformed data [28]. Correlations were calculated by Spearman-Rho-tests. The level of significance was set at α= 0.05 (two-tailed) for all tests.

## Results

For both studies ANOVAs on rank-transformed ratings were performed in order to estimate the impact of trial repetition. In both experiments ANOVAS with factors condition and repetition on VAS and NRS revealed no significant main effect of repetition and no significant condition-by-repetition interaction (all p > 0.86). Thus, the ratings were pooled across conditions by taking their median. Median perceived itch intensity before scratching was 28.75 on the VAS and 30.0 on the NRS in the mirror experiment, and 42.5 on the VAS and 40.0 on the NRS in the video experiment. There was no significant difference in perceived itch intensity between the experimental conditions prior to scratching. 

### Mirror experiment

**Figure 3:**
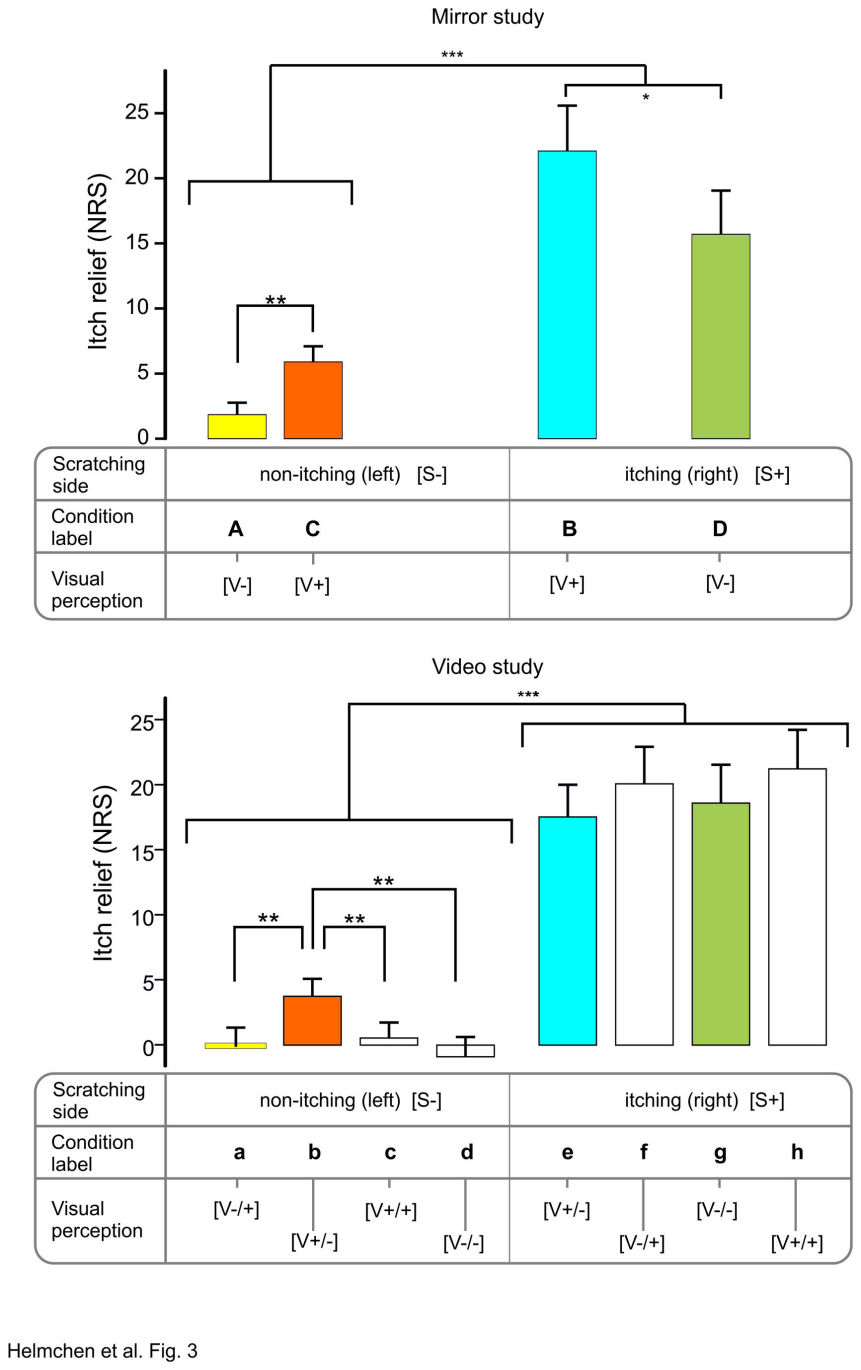
Comparison of condition-related itch relief in both experiments. Average rating difference (RD) of itch intensity (= itch relief) in the mirror (upper panel) and video experiment (lower panel) on the NRS (mean pre-post rating differences ± SEM). For better identification experimental conditions are labeled by capital (mirror experiment, A-D) and small (video experiment, a-h) letters. While the scratching side is shown above for the non-itching (left) [S-] and the itching right [S+] forearm, the visually perceived forearm is indicated below: visual percept that scratching is applied to the non-itching (left) forearm only [V-/+]; itching (right) forearm only [V+/-], to both forearms [V+/+], or visual percept that no scratching is applied [V-/-] (see also Table 1). In the case of two visible forearms, one forearm is flipped (mirrored), the other is visible as it is normally seen. Corresponding conditions in the mirror and video experiment are highlighted by the same colors (see also Table 2). *=p<0.05; **=p<0.01, ***=p<0.005

In the mirror experiment, itch relief differed significantly across the four conditions, both on the NRS (d.f. = 3, Chi^2^ = 40.0, p < 0.001, Figure 3] and on the VAS (d.f. = 3, Chi^2^ = 28.3, p < 0.001). Itch relief, as expected, was significantly stronger in the two conditions in which scratching was applied at the itching (right) side (*S+*; Figure 1B, D) than in the two conditions in which scratching was applied to the non-itching (left) side (*S*-, Figure 1A, C). Interestingly, scratching at the itching (right) side elicited a larger itch relief under visuo-tactile congruency [S+V+] (Figure 1B) than under visuo-tactile incongruency [S+V-] (Figure 1D). Critically, when scratching was applied to the non-itching (left) forearm, itch relief was significantly greater when the non-itching (left) forearm was visually perceived as the itching (right) forearm [*S-V+*] (NRS: 30, VAS: 36.8%, Figure 1C) then when the non-itching (left) forearm was perceived as the non-itching (left) forearm [S-V-] (NRS: 20%, VAS: 22.2%, Figure 1A). 

**Table 2:** Correspondence of experimental conditions in experiment 1 (A-D) and experiment 2 (a-h).

Scratching	Experimental condition	
Right (itching) forearm [S+]	B (e)	D (g)
Left (non-itching) forearm [S-]	A (a)	C (b)

### Video experiment

As in the mirror experiment, itch relief in the video experiment was significantly stronger in the conditions in which scratching was applied at the itching (right) side, both for VAS (18.5 ±2.7, mean of all 4 conditions e-h, Figure 2, Table 1) and NRS (18.8 ±2.6) vs. VAS (1.1 ±1.2) and NRS (0.7 ±1.2; mean of all 4 conditions a-d, Figure 2, Table 1), respectively (VAS: Z = 3.7, p< 0.001; NRS: Z = 3.8, p< 0.001). Interestingly, conditions S+ [V-/+] and S+ [V+/+] (Figure 2 f,h) showed a trend to stronger itch relief than condition S+ [V-/-] and S+ [V+/-] (Figure 2 e, Figure 3 lower panel). The latter was expected to elicit strongest itch attenuation as it provides visuo-tactile congruency at the itching site (“seeing and perceiving scratches where it itches”: S+ [V+/-]). More importantly, when scratching was applied to the non-itching (left) forearm, itch relief was strongest [(NRS: 17.9% (median), VAS: 26.1% (median) (Figure 2b, Figure 3 lower panel, b)] when the non-itching (left) forearm was visually perceived as the itching (right) forearm S- [V+/-], as compared to all other conditions with left-sided stimulation (<4%) (Figure 3). Thus, itch relief in this condition S- [V+/-] corroborated the findings in the mirror experiment S- [V+] (Figure 1C).

## Discussion

The current study tested the hypothesis that “mirror scratching”, i.e., scratching a mirrored non-itching forearm, can attenuate a circumscribed, experimentally induced itch. In line with our hypothesis, we observed a significant attenuation of itch by remote scratching only in an experimental condition in which the participant visually perceived the forearm being scratched as the itching forearm. Observing the non-itching forearm being scratched without the visual illusion did not result in itch reduction.

### Central mechanisms of itch relief

Itch is an annoying and unpleasant but usually not painful sensation which evokes the desire to scratch. The urge to scratch is reflected in activations of brain regions engaged in the processing of the sensory and affective / motivational aspects of itch and premotor cortical areas involved in the action preparation [29-32]. Limbic, ventral prefrontal and ventral striatal activations are associated with the desire to achieve itch relief by scratching [31,33]. Ventral prefrontal cortex generates reward predictions and ventral striatum activation is associated with motivational processing [34]. Scratch-related activations may reflect the - for a short term - highly rewarding nature and often addictive behavioural response of scratching [11]. Itch-related activation in the anterior cingulate cortex [29,31,33] is reduced during scratching which probably reflects inhibition of itch processing [35]. Mechanical pinprick stimuli can reduce itch intensity not only when they are applied adjacent to the itching skin site but also when they are applied at the contralateral extremity [36]. Taken together, these findings strongly support the notion that itch relief is partly under control of central neural processes. However, in the current study, scratching at a remote site alone did not result in itch relief. Significant itch relief by remote scratching was observed only if the participant visually perceived the limb being scratched as the itching limb. This is in line with the observation that some phantom itch patients have reported phantom itch attenuation by observing scratching of their intact foot through a mirror [13].

### Perceptual congruency and mismatch of multisensory stimulation

In the current study, itch relief was strongest when the itching forearm was actually being scratched [S+], irrespective of the visual percept. This is in line with previous studies and underlines the predominant role of tactile-pruriceptive congruency (“feeling being scratched where it itches”) in itch relief. However, moderate but significant itch relief was also observed in conditions with tactile-pruriceptive incongruence [S-], when the itching forearm was visually perceived as being scratched [V+] (“seeing being scratched where it itches”). Because in conditions with tactile-pruriceptive incongruence itch relief is unlikely to be due to peripheral mechanisms, these findings provide further evidence that central mechanisms play a pivotal role in itch relief and that itch relief is partly independent of somatosensory input from the periphery. This raises the question what exactly led to the significant itch relief observed under tactile-pruriceptive incongruence in the current study?

One possibility is that visual-tactile enhancement might play a role in itch relief. Previous studies indicate that visibility of touch stimulation can improve tactile perception [37,38]. Thus, one might speculate that particularly in the first experiment, where visibility of the non-itching forearm was not completely controlled, visual perception of being scratched at both forearms in combination with actually being scratched at the non-itching forearm led to visuo-tactile enhancement which in turn led to itch relief although the itching site was in fact not scratched. However, this possibility is ruled out by the second experiment. Intriguingly, in that experiment we observed significant itch relief *only* when the participant visually perceived solely the itching forearm as being scratched (S-V+/-). The visual percept that both forearms were being scratched (S- V+/+) did not lead to significant itch relief. This provides some evidence that itch relief under tactile-pruriceptive incongruence in the current study was due to the visual illusion that the itching forearm was being scratched (i.e. “mirror scratching”) and not simply to visuo-tactile enhancement. 

Bodily illusions can result from conflicting or ambiguous multisensory information [14]. Self-attribution of sensory stimuli to body limbs has been suggested to be mediated by multisensory perceptual interactions [39] which may, for example, be accomplished by (i) mirror images [15] or mirrored visual feedback [25] of body limbs with conflicting visuo-tactile information or (ii) simultaneous stimulation of an own and artificial limb, e.g. in the rubber hand illusion. 

Observing a limb in a mirror can lead to the illusion that the mirrored limb is the contralateral limb if the mirror is oriented in a way that the mirrored limb is in place of the contralateral limb. A mirror box, placed vertically on the table in front of a subject’s hand, has been used to elicit synaesthesia [15]. Patients with phantom limb perceptions placed their normal hands into a mirror box and were asked to look into the mirror, thus creating the illusion of observing two hands, while in fact they saw only the mirrored image of the normal hand. When the normal hand was touched while they were seeing its mirrored reflections they noticed tactile, i.e. referred sensations on the phantom hand. This intermanual referral of tactile sensations was crucially dependent on visibility of the mirrored image. However, our experiments differed from mirror box studies [15] such that itch attenuation was only seen when the participant visually perceived solely the itching forearm as being scratched [S-[V+] in the mirror experiment,) and S- [V+/-] in the video experiment] but not when both forearms were visible. 

Therefore we suggest that the scratching sensation in our study is probably transferred to the visually perceived itching limb (although the non-affected limb was physically scratched) and contributed to itch relief.

For several reasons mechanisms of itch relief by mirrored visual feedback should not be confused with another bodily illusion that has been extensively studied during recent years, i.e. the rubber hand illusion [39-41]. In this illusion, a person observes a rubber hand being touched in synchrony with touches applied at their own, but visually hidden hand. Once the rubber hand is spatially orientated as the person’s real hand, simultaneous stimulations at corresponding body sites can create the illusionary perception that the rubber hand belongs to one’s own body. This illusion can be quite strong including, e.g., the perception of fear when the rubber hand is under threat [42]. First, we used a mirror and video-mediated mirrored images to elicit visual illusions and to change sensory perceptions. Second, itch relief was not achieved by simultaneous tactile stimulations (no effect on observing both forearms). Third, itch attenuation does not result from simultaneous stimulation of two (real and rubber) hands lying in close vicinity and similar spatial orientation with respect to one’ own bodily references but it occurs despite the fact that tactile stimulation (scratch) is opposite to the itching limb. We believe that the effect requires that the participants visually perceived their mirrored non-itching hand as their right itching hand which is visually perceived as being scratched. Therefore, the mechanisms of mirror visual feedback by intersensory perceptual interactions are clearly different from the rubber hand illusion. 

All in all, we suggest that itch relief by “mirror scratching” as observed in the current study may result from inappropriate integration and weighing of simultaneous, spatially coded, multisensory (visual, tactile, pruriceptive) signals leading to a transient intersensory perceptual congruency of visual, tactile and pruriceptive signals. However, while both effects result from an inappropriate integration of signals, the particular neural algorithms underlying these misperceptions might be distinctly different. The pattern of itch relief observed in the current study suggests that multisensory visuo-tactile-pruriceptive integration in itch perception is governed by a weighing process: tactile-pruriceptive congruency (“feeling being scratched where it itches”) led to stronger itch relief than visuo-pruriceptive congruence (“seeing being scratched where it itches”). Exploring the neural mechanisms that underlie this weighing will be a challenging task for future studies.

### Potential clinical role

Itch is a prevalent symptom of allergic and inflammatory skin disease. Patients must not scratch itching skin sites to prevent deterioration. Conventional drug therapy of pruritus does often not lead to satisfactory itch relief. The current study provides evidence that partial itch relief might be achieved by “mirror scratching”, i.e., by creating the illusionary visual perception that an itching limb is being scratched while in fact non-lesional skin of the contralateral limb is being scratched. The use of illusionary visual mirror feedback has successfully been used to partially restore brain function: e.g. in after stroke [17,18,43], in complex regional pain syndrome [44,45], and in phantom pain [46-51]. Technically, a related [25] and our study have shown that mirroring can be effectively modulated by video-mediated images allowing better applicability in the clinical context. Modulation of activity in itch-related brain areas by mirror therapy might help to counteract maladaptive functional and/or structural cortical reorganization. For example, central itch-related neural processing considerably differs between lesional and non-lesional skin [52]. In atopic eczema, for example, deactivation of itch-processing brain regions might be impaired leading to a deficient capacity to suppress itch perception. Future studies will show whether this can be counteracted by a “mirror scratching therapy”.

## Conclusions

We demonstrated that relief of an experimentally elicited, circumscribed itch can be achieved by scratching the non-itching limb the visual illusion is created that the itching limb is being scratched. This effect probably results from transient inter-sensory perceptual congruency of visual, tactile and pruriceptive signals. “Mirror scratching” might have considerable clinical impact as it could help to reduce itch perception in focal skin diseases with unbearable pruritus.
